# An assessment of Nigeria’s microlivestock value chain: insights from six species

**DOI:** 10.1007/s11250-026-04960-8

**Published:** 2026-03-14

**Authors:** Dolapo Enahoro, Oladeji Bamidele, Dare Akerele, Olusegun O. Ojebiyi, Adetunji O. Iyiola-Tunji, Wasiu A. Olaniyi, Joseph Karugia, Isabelle Baltenweck

**Affiliations:** 1 People, Policies and Institutions Program, International Livestock Research Institute, C/o IWMI-Ghana, Accra, Ghana; 2 Department of Biological Sciences, Kings University, Odeomu, Osun State Nigeria; 3 People, Policies and Institutions Program, International Livestock Research Institute, Nairobi, Kenya; 4 Department of Agricultural Economics and Farm Management, Federal University of Agriculture, Abeokuta, Nigeria; 5 Department of Animal Nutrition and Biotechnology, Faculty of Agricultural Sciences, Ladoke Akintola University of Technology, Ogbomoso, Oyo State Nigeria; 6 National Agricultural Extension and Research Liaison Services (NAERLS), Ahmadu Bello University, Zaria, Kaduna State Nigeria; 7 Department of Animal Science, Adekunle Ajasin University, Akungba-Akoko, Ondo State Nigeria

**Keywords:** Livelihoods, Livestock development, Gender, Youth, Minilivestock, Policy

## Abstract

Microlivestock farming offers potential to improve incomes and nutrition in Nigeria, yet the subsector remains fragmented and underdeveloped. There is, in addition, a lack of reliable data to support value-chain-wide development. This study presents an explorative, mixed-methods assessment of the value chains of six microlivestock species commonly raised in Nigeria. The study was conducted in six purposively selected states: Oyo (rabbits), Anambra (grasscutters), Ondo (snails), Katsina (guinea fowl), Plateau (quail), and Kwara (honeybees), spanning five agroecological zones, integrating data from questionnaire surveys (*n* = 114), gender-disaggregated focus group discussions (*n* = 120), and key informant interviews (*n* = 18). Microlivestock production was the primary source of livelihood for 10% (honeybees) to 33.3% (grasscutter) of farmers sampled. Across the value chains, the proportions of surveyed farmers engaged in multiple economic activities ranged from 10.0% (guinea fowl) to 88.9% (quail), indicating the supplementary role of microlivestock to household incomes. Farmer association membership was highest among rabbit (95%), honeybee (70%), and grasscutter (61.1%) farmers. In comparison, farmers of guinea fowl (10.0%), quail (17.0%), and snail (27.8%) in the sample had lower rates of association membership. The farmers reported persistent challenges, including limited access to specialized feed and veterinary services, and weak market linkages. The study participants also reported gender disparities and barriers to youth participation in microlivestock value chains, despite high youth interest. The study identified strategic opportunities in the formation of cooperatives, digital marketing, e-commerce, species-specific extension, and supporting youth-led enterprises. The findings underscore the need for gender-sensitive, youth-inclusive policies and programs, alongside species-targeted interventions in the subsector. This assessment provides evidence to support current livestock policies and strategies, as well as the integration of microlivestock into broader livestock reform agendas.

**Supplementary Information:**

The online version contains supplementary material available at 10.1007/s11250-026-04960-8.

## Introduction

The consumption, production, processing, and trade of microlivestock, which are also known as “minilivestock”, and include rabbits, snails, grasscutters, guinea fowl, honeybees, and quail, offer promising pathways for improving nutrition, diversifying livelihoods, and promoting environmentally sustainable food systems, especially among smallholder households in resource-poor, low external-input production communities of sub-Saharan Africa (Udo and Cornelissen 1998; Payne and Wilson 1999; FAO 2001; Ingweye and Kalio 2020; Ayeni et al. 2023). Since the first livestock census in Nigeria over three decades ago, microlivestock has represented a rapidly growing niche in Nigeria’s livestock sector (FAO 2001). These species are valued for their short production cycles, ease of management, minimal land and labour requirements, suitability for urban and peri-urban environments, compatibility with women and youth-led enterprises, and potential for income generation and export (Assan 2017; Klapwijk et al. 2020; Imoru and Babadipe 2019). Despite the stated advantages, the microlivestock subsector remains underdeveloped, mainly with rudimentary production technologies, weak input and output markets, and limited value chain integration. Further, there is limited up-to-date information to support coordinated public or private sector interventions towards the sector’s development.

The recent establishment (in 2024) of the Federal Ministry of Livestock Development (FMLD) in Nigeria has brought increased attention to various livestock sub-sectors, including microlivestock, with one of the mandates of the new ministry being to promote the development of microlivestock alongside traditional ruminant and monogastric species (FMLD 2025). Similarly, a presidential livestock reforms implementation committee identified microlivestock (particularly rabbits, grasscutters, snails, and quail) as a priority sub-sector for interventions towards inclusive transformation of the livestock sector (FRN 2024). The National Agricultural Technology and Innovation Policy (NATIP) 2022–2027, which serves as the overarching policy framework for livestock development in Nigeria, and more recently, the Nigeria Livestock Growth Acceleration Strategy (NL-GAS) 2025–2030, identify grasscutter, honeybee, snail, and rabbit as key value chains for strategic development (FMARD 2022; Nkwocha 2025). However, these policy signals lack the support of up-to-date assessments of the microlivestock value chains in the country, which are needed to design concrete programs and projects for microlivestock development. Existing national assessments (FMARD and The World Bank, 2020) and scholarly publications (mainly quantitative) are limited in scope, outdated, or fragmented across species and regions. Evidence on gender roles, youth participation, marketing systems, and value addition is particularly scarce.

This exploratory study aimed to provide an updated assessment of relevant production, processing, marketing, and related trends in six microlivestock value chains (rabbit, grasscutter, honeybee, snail, guinea fowl, and quail). It also sought to complement existing information on these animal value chains, primarily through capturing the experiences and perceptions of microlivestock value chain actors and subject matter experts. Findings from this study should be relevant for generating recommendations that guide the development of inclusive policies and the design of targeted investments for (micro) livestock development in Nigeria.

## Materials and methods

### Study design and scope

This study employed a mixed-methods approach combining qualitative and quantitative data to characterise the structure, performance, and constraints of six microlivestock value chains in Nigeria. The focal species are rabbits (*Oryctolagus cuniculus*), grasscutters (*Thryonomys swinderianus*), honeybees (*Apis mellifera*), snails (*Archachatina marginata*), quails (*Coturnix coturnix japonica*), and guinea fowl (*Numida meleagris*). These species were selected based on their inclusion in national livestock development frameworks (FMARD 2022) and validated during two (2) stakeholder workshops held in the Federal Capital Territory (FCT), Abuja in July and December of 2023.

Research approval was obtained from the Institutional Research Ethics Committee (IREC) of the International Livestock Research Institute (ILRI-IREC2024-027), and the Ethics Committee of the Redeemer’s University (RUN/REC/2024/198). All participants in the study provided informed consent.

### Field sites and sampling

**Fig. 1:**
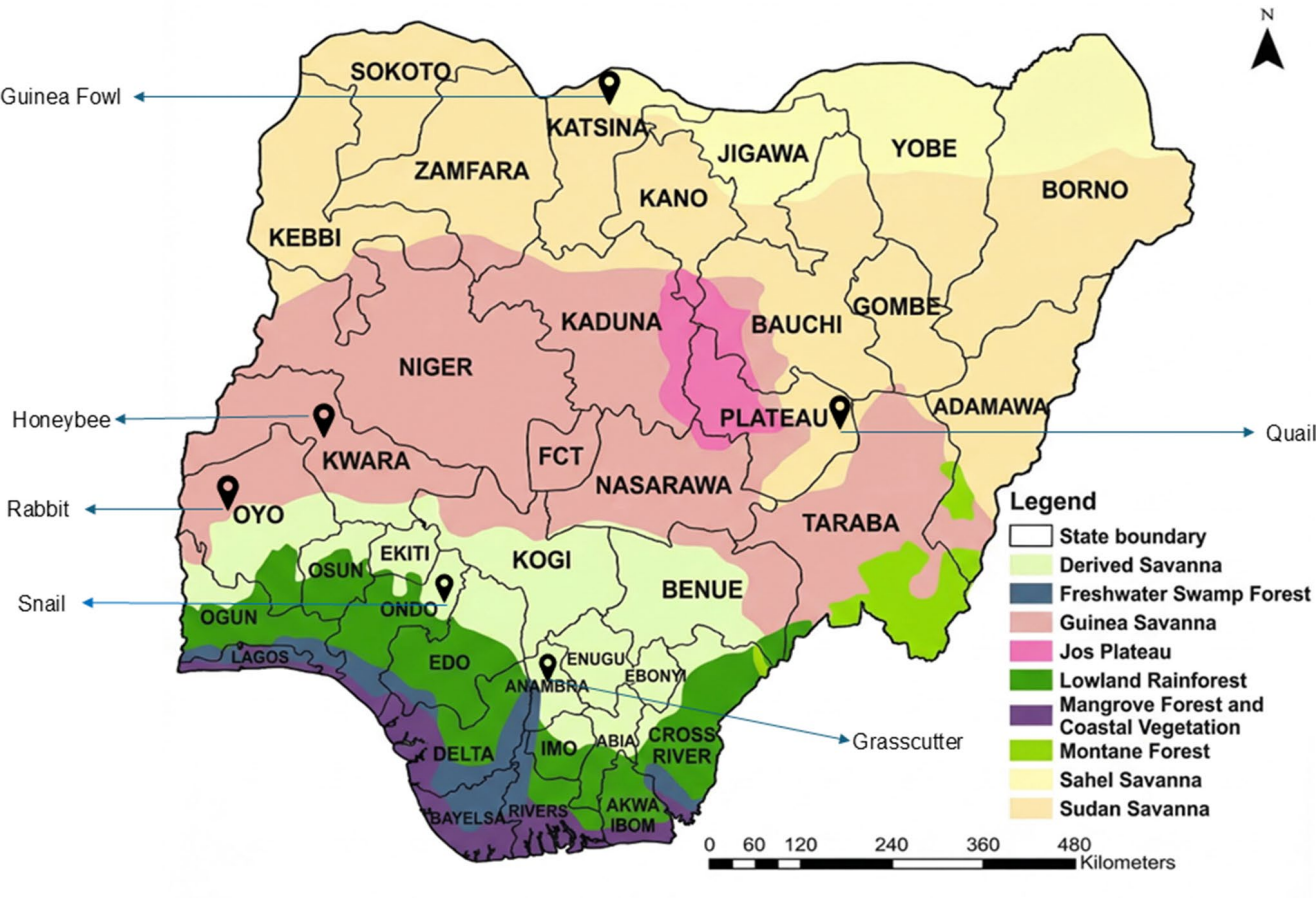
Selected study locations for the microlivestock species. (Source of agroecological map: FORMECU, 1998)

Primary data were collected between August and September 2024 from locations in six states representing agroecological and species-specific production hubs: Oyo (rabbits), Anambra (grasscutters), Kwara (honeybees), Ondo (snails), Plateau (quails), and Katsina (guinea fowl) (Fig. 1; Table 1). In addition to these being states with high levels of microlivestock farming activity and/or active producer associations for the various microlivestock value chains (signifying high levels of commercial activity and the existence of production clusters), the selection of sites was informed by consultations with representatives from research and extension institutions, and with the animal value chain desk officers at FMLD (Afolabi 2023; Ajao et al. 2014; Aminu et al. 2017; Folorunso et al. 2018; Okeke and Oruh 2020; FMARD 2022; Ayeni et al. 2023). For each species and location, a purposive sample of 20 producers (10 males and 10 females) was selected. In total, 120 microlivestock farmers participated in the focus group discussions, of which 114 further completed the questionnaire surveys. Two gender-disaggregated (one male and one female) focus group discussions (FGDs) were conducted per site, resulting in 12 FGDs overall. In addition, three key informant interviews (KIIs) per species were conducted with a national association representative, a state-level stakeholder, and a subject matter expert (*n* = 18 KIIs).

**Table 1:** Agroecological and demographic features of the selected states

Features	Katsina	Kwara	Plateau	Oyo	Anambra	Ondo
Agro-ecological zones	Sudan to Sahel savannah	Guinea savannah	Montane + Sudan/ Guinea savannah	Guinea savannah to Derived savannah	Derived savannah	Rainforest to Derived savannah
GPS coordinates (approximate)	12.3797° N, 7.6306° E	8.9669° N, 4.3874° E	9.2182° N, 9.5179° E	8.1574° N, 3.6147° E	6.2209° N, 6.9370° E	7.0210° N, 5.0568°E
Land area (Km^2^)	23,561	35,705	27,147	26,500	4,865	15,500
Human population	10,368,483	3,551,023	4,717,305	7,976,081	3,592,163	5,316,603
Youth (%) (15–29)	26.4	29.9	28.0	24.0	23.4	24.9
Temperature (°C) (min- max)	21.3–34.5 °C;	22–37 °C	16–28 °C	23.2–31.7 °C	23.7–32.8 °C	26.8–28.8 °C
Rainfall (mm per annum) (min – max)	640–1056 mm	990.3 -1,318 mm	1317–1460 mm	1,050 mm − 1,350 mm	1520–2020 mm	1440–1711 mm
Seasons	Wet: May – September; Dry: October – April; Harmattan: December-February	Wet: April-September; Dry: November - March	Wet: April – October; Dry: November- March; Harmattan: December-February	Wet: April-September; Dry: November - March	Wet: April – October; Dry: November-March	Wet: April – October; Dry: November-March

Sources: Akinyemi et al. 2013, 2021; Ifeka and Akinbobola 2015; Eludoyin et al. 2016; NBS 2016, 2020; Nnadi et al. 2019; FAO 2020; NPC 2020; Faweya et al. 2023; www.Latitude.to; www.weatherandclimate.com; www.Kipdeco.com

### Data collection instruments

Structured questionnaires were designed and adapted from previous studies on rabbit (Baruwa 2014; Oluwatusin 2014), grasscutter (Arowolo et al. 2021; IAR&T 2021; Uka et al. 2021), honey bee (Ejikeme and Ugwu 2023; Adeokun et al. 2022), snail (NAERLS 1995; Baba and Adeleke 2006; Aiyeloja and Ogunjinmi 2010), guinea fowl (NAERLS 2004; Yakubu et al. 2014; Baruwa and Sofoluwe 2016), and quail (Saka et al. 2018; Gbadamosi et al. 2024) value chains. The survey instrument was implemented as species-specific questionnaire modules, with respondents completing the module relevant to their enterprise. The number of questions, therefore, varied slightly by species: rabbit (30), grasscutter (29), honeybee (28), snail (28), guinea fowl (30), and quail (33). Across species, each module followed a common structure, organised into five sections: socio-demographic characteristics and livelihood diversification; input supply and service access; production systems and productivity parameters; products, value chain roles, market channels and prices; and awareness of relevant policies and institutional support. Question formats included binary (yes/no), categorical and ordinal responses, multiple-response items, and numeric entries (e.g. stock size, production volumes, prices, and durations). Multiple-response items were coded as binary indicators for analysis, ordinal variables were retained in ranked form, and numeric variables were analysed as continuous measures. The complete questionnaire modules are provided in Online Resource 1.

FGD and KII guides (Online Resource 2) were based on a toolkit developed for livestock value chain assessments under the CGIAR Research Program on Livestock (Baltenweck et al. 2019). The guides were designed to capture qualitative insights on production constraints, opportunities, gender roles, youth participation, market access, value addition, and institutional support across microlivestock value chains. FGDs were conducted separately for men and women to enable gender-disaggregated analysis. Before deployment, all field officers were trained on the data collection tools and effective facilitation of gender-disaggregated FGDs. The tools were pilot tested by the team of trained field officers with prior experience in both qualitative and quantitative livestock research. The team included a lead researcher (facilitator), two research assistants (note-taker and co-facilitator), and a community mobiliser fluent in the local language. The survey tools were interviewer-administered. Data from the questionnaire survey was collected on smartphones using Google Forms. FGDs and KIIs were recorded using an audio recording software application on smartphones, along with detailed notetaking.

### Data analysis

The quantitative data obtained from the surveys were downloaded from Google Cloud Services, cleaned in Microsoft Excel (version 16.0), and analysed using SPSS (version 27). Descriptive statistics were used to summarise participants’ responses on producer characteristics, production parameters, market access, and financial outcomes. Financial data, such as prices, were standardised using the prevailing foreign exchange rate during the study period (₦1,613.55 = US$1.00, as of August 2024, Central Bank of Nigeria). Qualitative data from FGDs and KIIs were manually transcribed, reviewed for clarity, and thematically coded using a combination of deductive (predefined themes) and inductive (emergent) approaches with the aid of ATLAS.ti (Version 23). Coding was triangulated with survey findings and literature to enhance internal validity and depth of interpretation. All data have been anonymised to protect participant identity. Key themes were categorised under four cross-cutting dimensions: Gender roles, Youth interest, Market access, and Value addition. To visualise these intersections, a relationship map was developed using the NetworkX library (Version 3.5) in Python (Version 3.12.6) (Hagberg et al. 2008; Python Software Foundation 2024). Findings from all sources (survey data, FGDs, KIIs, and literature) were synthesised to inform species-specific profiles and derive cross-cutting recommendations.

## Results

Given the exploratory design and modest sample sizes, the study’s findings are interpreted as indicative patterns deriving from the study sample, rather than as statistically representative data that would define the broader microlivestock farming population in Nigeria.

### Demographic characteristics of microlivestock farmers

The demographic profiles of the respondents sampled in this study are presented in Table 2. Among the sampled producers, microlivestock farming was most practised by individuals aged 36 ± 6.5 (honeybees) to 43 ± 9.0 (snails) years. Most respondents were married and had a tertiary education, except guinea fowl farmers, 95% of whom reported no formal education. A third or less of respondents in the study sample were solely engaged in microlivestock production. This ranged from 10% (honeybee) to 33.3% (grasscutter).

**Table 2:** Demography of study participants

Species	*N*	Age (years) Mean ± standard deviation	Female (*n*, %)	Single (*n*, %)	Widow/widower (*n*, %)	Married (*n*, %)	No Formal Education (*n*, %)	Secondary Education (*n*, %)	Tertiary Education (*n*, %)	Sole Livelihood Source (*n*, %)
Rabbit	20	43.0 ± 11.1	9 (45.0%)	1 (5%)	0 (0.0%)	19 (95.0%)	0 (0.0%)	2 (10.0%)	18 (90.0%)	4 (20.0%)
Grasscutter	18	42.0 ± 9.7	3 (16.7%)	3(16.7%)	0 (0.0%)	15 (83.3%)	0 (0.0%)	4 (22.2%)	14 (77.8%)	6 (33.3%)
Honeybee	20	36.0 ± 6.5	10 (50.0%)	4(20.0%)	0 (0.0%)	16 (80.0%)	0 (0.0%)	1 (5.0%)	19 (95.0%)	2 (10.0%)
Snail	18	43.2 ± 9.0	12 (66.7%)	3 (16.7%)	0 (0.0%)	15 (83.3%)	2 (11.1%)	4 (22.2%)	12 (66.7%)	3 (16.7%)
Quail	18	41.1 ± 8.7	8 (44.4%)	1 (5.6%)	0 (0.0%)	17 (94.4%)	0 (0.0%)	1 (5.6%)	17 (94.4%)	3 (16.7%)
Guinea Fowl	20	47 ± 10.3	10 (50.0%)	0 (0.0%)	4(20.0%)	16 (80.0%)	19 (95.0%)	1 (5.0%)	0 (0.0%)	6 (30.0%)

N = total number of respondents per species; n = number of respondents in category (percentages in parentheses)

### Ownership of other livestock species

The highest co-ownership of traditional livestock species was reported for poultry, with nearly 60% of all microlivestock farmers surveyed reporting that they (also) raised chicken/poultry (Table 3, multiple responses were allowed; hence, percentages do not total 100). Goats were the second most kept livestock species among the microlivestock farmers, with 21% of the sampled microlivestock farmers raising goats. Notably, 50% of guinea fowl farmers sampled in this study kept cattle, which was more than any other group of microlivestock farmers.

**Table 3:** Other livestock species kept by study participants*

Other livestock species reared	Rabbit (*n*,%)	Grasscutter (*n*,%)	Honeybee (*n*,%)	Snail (*n*,%)	Quail (*n*,%)	Guinea Fowl (*n*,%)
Goat	3 (15.0%)	2 (11.1%)	3 (15.0%)	4 (22.2%)	6 (35.3%)	7 (35.0%)
Sheep	1 (5.0%)	0 (0%)	2 (10.0%)	0 (0%)	3 (17.6%)	0 (0%)
Cattle	0 (0%)	0 (0%)	0 (0%)	0 (0%)	2 (11.8%)	10 (50.0%)
Pig	0 (0%)	3 (16.7%)	0 (0%)	1 (5.5%)	1 (5.9%)	0 (0%)
Chicken / Poultry	15 (75.0%)	7 (38.9%)	8 (40.0%)	11 (61.1%)	10 (55.6%)	17 (85.0%)
Fish	1 (5.0%)	0 (0%)	1 (5.0%)	1 (5.5%)	0 (0%)	0 (0%)
Duck	0 (0%)	0 (0%)	0 (0%)	1 (5.5%)	0 (0%)	0 (0%)

*Total possible number of farmers for each microlivestock type is 20, see Table 2; n = number of respondents in category (percentages in parentheses)

### Production and husbandry practices

Key production characteristics of the surveyed microlivestock producers are presented in Table 4. The table excludes information on honeybees’ production, which has somewhat different characteristics. The micro-livestock farmers reported significant variation in stock size, reproduction, maturity and management. Snails and quails have the highest average stock sizes (400 and 350, respectively) while grasscutters have the lowest (11). As per their biology, quails and guinea fowls lay a high number of eggs per year (84 and 79), compared to rabbits and grasscutters, which have moderate litter sizes (of 4 to 5) at birth. Further, rabbits and quails mature fastest, while grasscutters and guinea fowls reach the highest market weights. Housing practices vary widely, with rabbits and grasscutters mostly cage-housed, and guinea fowls primarily floor-raised. Commercial feed is most used with quails, while guinea fowls rely on scavenging. Breeding stock sources ranged from commercial farms to informal networks, depending on species.

**Table 4:** Production and husbandry practices across five microlivestock species

Variable	Rabbit	Grasscutter	Snail	Quail	Guinea Fowl
Average stock size (mean ± Standard deviation (SD))	35.0 ± 6.2	11.0 ± 2.7	400.0 ± 325	350.0 ± 200	21.0 ± 4.3
Litter size at birth /No of clutches/year/No of eggs per hen (mean ± SD)	7.0 ± 0.96	4.9 ± 1.2	3.5 ± 1.5	84.1 ± 8.4	78.5 ± 13.13
Litter size at weaning/clutch size (mean ± SD)	5.4 ± 1.31	4.4 ± 1.2	16.6 ± 11.0	2.71 ± 1.6	NA
Age (mean ± SD) at weaning/hatchling to Grower/Sexual maturity (days)	38.5 ± 9.0	45.5 ± 9.4	108 ± 78	46.6 ± 10.7	196 ± 21
Age (mean ± SD) at market weight (months)	3.5 ± 0.8	6.5 ± 0.5	7.3 ± 3.0	2.0 ± 0.5	5.0 ± 0.6
Market weight (g) (mean ± SD)	2200.0 ± 360.0	3900.0 ± 2400.0	180.0 ± 20.0	127.9 ± 15.2	819 ± 160
Housing type: Cage/pens System (n,%)	19 (95.0%)	18 (100.0%)	8 (44.4%)	1 (5.6%)	0 (0.0%)
Housing type: Floor system/baskets (n,%)	0 (0.0%)	0 (0.0%)	2 (11.1%)	15 (83.3%)	18 (90.0%)
Housing type: Mixed/drums+tyres (n,%)	1 (5.0%)	3 (16.7%)	10 (55.6%)	2 (11.2%)	2 (10.0%)
Use of commercial feed (n,%)	12 (60.0%)	10 (50.0%)	9 (50.0%)	15 (83.0%)	Low
Veterinary (para-vet, CAHW) access (n,%)	3 (15.0%)	4 (22.2%)	2 (11.1%)	16 (88.9%)	4 (20.0%)
Self-treatment (n,%)	17 (80.0%)	15 (83.3%)	16 (88.9%)	2 (11.1%)	16 (80.0%)
Main feed source	Commercial pellets, forages	Formulated feed, forage	Formulated feed, kitchen waste (vegetables, fruits), forages	Commercial feed	Scavenging, local grains, farm residues
Most common breeds/types	Exotic breeds (Hyla, Chinchilla, New Zealand), crossbreeds	Crossbreeds	*Archachatina marginata*	Japanese quail	Local breed (helmeted)
Primary source of Breeding stock	Commercial farms and associations	Commercial farms and associations	Association, and local market	Research institutes, and commercial hatcheries	Informal sources (inherited, gifts) and local market

NA: not applicable; n = number of respondents in category (percentages in parentheses)

Data from honeybee producers showed that most of the farmers operated on a small scale, with 70% managing fewer than 11 hives. The reported average yield per hive was 17.8 ± 9.8 L of honey per season. Wildflower honey (60%) and forest honey (55%) were the most common types produced. Respondents indicated significant variation in the time from hive installation to colonisation, as well as from colonisation to the first honey harvest. The reported annual production capacity varied widely, with 45% of producers producing less than 250 kg annually. Feeding practices included pollen substitutes (40%), sugar syrups (20%), and essential oils (20%).

### Market participation and functional roles across value chains

Market orientation varied considerably across the value chains (Table 5). Membership of producer associations was higher among rabbit (95%), honeybee (70%), and grasscutter (61.1%) farmers sampled, while guinea fowl (10.0%), quail (17.0%), and snail (27.8%) had lower participation. The marketing channels for farmers were mainly direct sales to consumers and restaurants/supermarkets, as well as through middlemen. Farmers predominantly engaged in the sale of live animals; however, value addition was more common among rabbit (40%), honeybee (50%), and quail (50%) producers, compared to grasscutter (11.1%), snail (22.2%), and guinea fowl (0%). The proportion of farmers involved in other commercial activities (e.g., processing) within the same value chain varied across the value chains. Quail (88.9%) and honeybee (85.0%) producers recorded the highest levels of multi-activity participation, followed closely by grasscutter (66.6%) and rabbit (55.0%) farmers. In contrast, lower levels of value chain activities were observed among snail (38.9%) and guinea fowl (10.0%) producers.

**Table 5:** Market and value chain characteristics by species

Variable	Rabbit	Grasscutter	Honeybee	Snail	Quail	Guinea Fowl
Members of producer association (n,%)	19 (95.0%)	11 (61.1%)	14 (70.0%)	5 (27.8%)	3 (17.0%)	2 (10.0%)
Farmers involved in multiple value chain activities (%)	11 (55.0%)	12 (66.6%)	17 (85.0%)	7 (38.9%)	16 (88.9%)	2(10.0%)
Live Product Sold (n,%)	20 (100.0%)	16 (88.9%)	Honey: 18 (90.0%)	16 (88.9%)	Birds: 13 (72.2%), Eggs: 14 (77.8%)	Birds: 18 (90.0%), Eggs: 20 (100.0%)
Processed Product Sold (n,%)	8 (40.0%)	2 (11.1%)	NA	4 (22.2%)	9 (50.0%)	0 (0.0%)
Other Products Sold (n,%)	Manure: 3 (15.0%)	Manure: 5 (27.8%), Sale of breeding stock: 9 (50.0%), Inputs: 3 (16.7%)	Beeswax: 14 (70.0%), Propolis: 4 (20.0%), Royal Jelly: 1 (5.0%), Honeycomb: 1 (5%)	Shells: 2 (11.1%), Inputs: 4 (22.2%)	Manure: 13 (72.2%), Inputs: 3 (16.7%)	Manure: 2 (10.0%)
Main Market Channels	Direct to consumer (95%), Middlemen (90%), Restaurants/supermarket (30%)	Direct to consumers (66.7%), Middlemen (22.2%), Restaurants/supermarket (27.8%)	Direct (90.0%), Middlemen (55.0%), Supermarkets (40.0%), Export (5%)	Direct to consumers (44.4%), Middlemen (38.9%), Restaurants/supermarkets (50.0%)	Direct to consumers (88.9%), Middlemen (55.6%), Restaurants/supermarkets (27.8%), researchers/project students (55.6%)	Direct to consumers (45.0%), Off-takers (55.0%)
Gender of primary buyer (Male, female or both)	Both (70.0%)	Males (50.0%)	Both (60%)	Females (44.4%)	Both (55.6%)	Males (95%)
Average Price (mean ± SD) of Primary Product (₦)	3,973 ± 1178.5/kg (live)	32,083 ± 13,993/live animal	6,375 ± 1,308 /litre (honey)	1,527.8 ± 681 /animal	Live bird: 732 ± 264 Eggs: 951 ± 348/dozen eggs	Live bird: 6,735 ± 1314

n = number of respondents in category (percentages in parentheses)

### Qualitative insights from focus group discussions and key informant interviews

FGDs and KIIs revealed participants’ perceptions of the behavioural, institutional, and gender-based dynamics that shape microlivestock value chains. These findings provide essential context for interpreting survey results and tailoring interventions to local realities.

### Gendered roles and constraints

Within the groups sampled, gender roles were sharply defined across all six species. Participants reported that men controlled most production and sales activities, while women were often restricted to support roles or processing. Female participants consistently cited market exclusion, limited land access, and lack of control over revenue.

Marketing of guinea fowl is limited to men alone… women in this community are most times not allowed to go to the market.

— Female participant (No. 07), Guinea Fowl FGD, Katsina State

My husband sells the rabbits, but I’m the one who feeds and cleans them.

— Female participant (No. 08), Rabbit FGD, Oyo State

### Youth participation: high interest, low support

In this study, young people were drawn to species with low capital and space requirements, such as rabbits, quail, and honeybees. However, they reported barriers related to start-up capital, equipment costs, and a lack of institutional support. Digital platforms provided market access, but youth lacked formal support for enterprise development and scaling.

Young people are interested in grasscutter farming, but the cages are too expensive.

— Male participant (No. 01), Grasscutter FGD, Anambra State

### Market access and seasonal vulnerability

Most producers relied on informal sales channels and faced sharp seasonal price fluctuations. Women, especially, reported being at the mercy of middlemen and price volatility.

Middlemen take advantage of us because there are no structured markets for rabbit meat.

— Female participant (No. 10), Rabbit FGD, Oyo State

There is high demand for guinea fowl only during Sallah (the major religious festival). In other periods, buyers are few and prices drop.

— Male participant (No. 08), Guinea Fowl FGD, Katsina State

### Barriers to value addition

Value addition was widely recognised as essential but rarely practised due to infrastructure and training gaps. Gender disparities were also apparent. This aligns with the quantitative results, which show that value-added product marketing was limited in most chains.

We want to make more money from rabbit farming, but we don’t have the electricity or freezers to store processed meat.

— Female participant (No. 10), Rabbit FGD, Oyo State

There’s no specialised equipment to process quail meat. The animal is small-sized, which makes it difficult to use the equipment that is available (for chicken), but we can still add value if we are trained.

— Male participant (No. 02), Quail FGD, Plateau State

### Prioritised challenges and opportunities: a gendered perspective

Table 6 presents the consensus ranking of key constraints and opportunities identified during FGDs, which were conducted separately for each species and disaggregated by gender.

**Table 6:** Gendered constraints and opportunities by species

Species	Gender	Challenge (Ranked)	Opportunity (Ranked)
Rabbit	Male	1. Limited market access	1. High utility of manure as a fertiliser
		2. Difficulties moving into processing operations	2. High demand from urban consumers
		3. High input costs	3. Rabbits can be raised in a small space
	Female	1. Poor market access	1. Feeds are readily available
		2. Shortages of capital	2. High utility of manure as a fertiliser
		3. Difficulties faced in adding value	3. High interest and support from the government and NGOs
Grasscutter	Male	1. Feed unavailability (dry season)	1. High market demand
		2. Veterinarians are not trained to manage grasscutters	2. Forages can be obtained at low cost
		3. High capital needs	3. An emerging niche market that youth can serve
	Female	1. Shortages of capital	1. Presence of training centres locally
		2. Limited access to veterinary services	2. An emerging niche market that youth can serve
		3. High input and low product prices	3. Potential for broad market reach through social media
Honeybee	Male	1. Proliferation of the market with substandard and adulterated products	1. Export markets are available and accessible
		2. Production equipment is scarce and expensive	2. High demand for by-products (wax, balm)
		3. High costs of packaging materials	3. High demand for honey-based products for medicinal and beauty purposes
	Female	1. Proliferation of the market with substandard and adulterated products	1. Export markets are available and accessible
		2. High risks of wildfires and vegetation change due to climate change	2. High demand for by-products (wax, royal jelly)
		3. High costs of packaging	3. Ease to participate in different activities in the value chain (e.g., by-products processing)
Snail	Male	1. High feed costs	1. High demand for by-products (e.g., snail oil, snail mucin)
		2. High rates of theft of snails	2. An emerging niche market that youth can serve
		3. High mortality rates of exotic breeds	3. Growing customer demand for leaner proteins
	Female	1. High feed costs	1. Low labour requirements that ease participation
		2. High levels of price instability	2. The availability of ready markets and marketing channels
		3. Low supplies of appropriate facilities/materials	3. Increasing consumer demand
Quail	Male	1. High mortality rates of young birds	1. Increased nutrition awareness among consumers
		2. Unavailability of feeds tailored to the needs of quails	2. An emerging niche market that youth can serve
		3. Low acceptability/awareness among most consumers	3. Potential for broad market reach through social media
	Female	1. Low consumer demand	1. Ease of rearing
		2. Small egg/meat sizes	2. Potential for broad market reach through social media
		3. Exclusion of quail from extension coverage	3. Potential to reach consumers with packaging and branding
Guinea Fowl	Male	1. Poor hatchability of eggs	1. High demand for cultural/traditional purposes
		2. Highly seasonal production and demand	2. High utility of manure as a fertiliser
		3. Low rates of literacy among the farmers	3. An emerging niche market that *male* youth can serve
	Female	1. Cultures and norms that exclude women from participating in grasscutter markets	1. High utility for home consumption
		2. Limited opportunities to get reasonable prices	2. High local demand for use as gifts
		3. High rates of theft of birds	3. High utility of manure as a fertiliser

### Visualising gendered priorities across microlivestock species

**Fig. 2:**
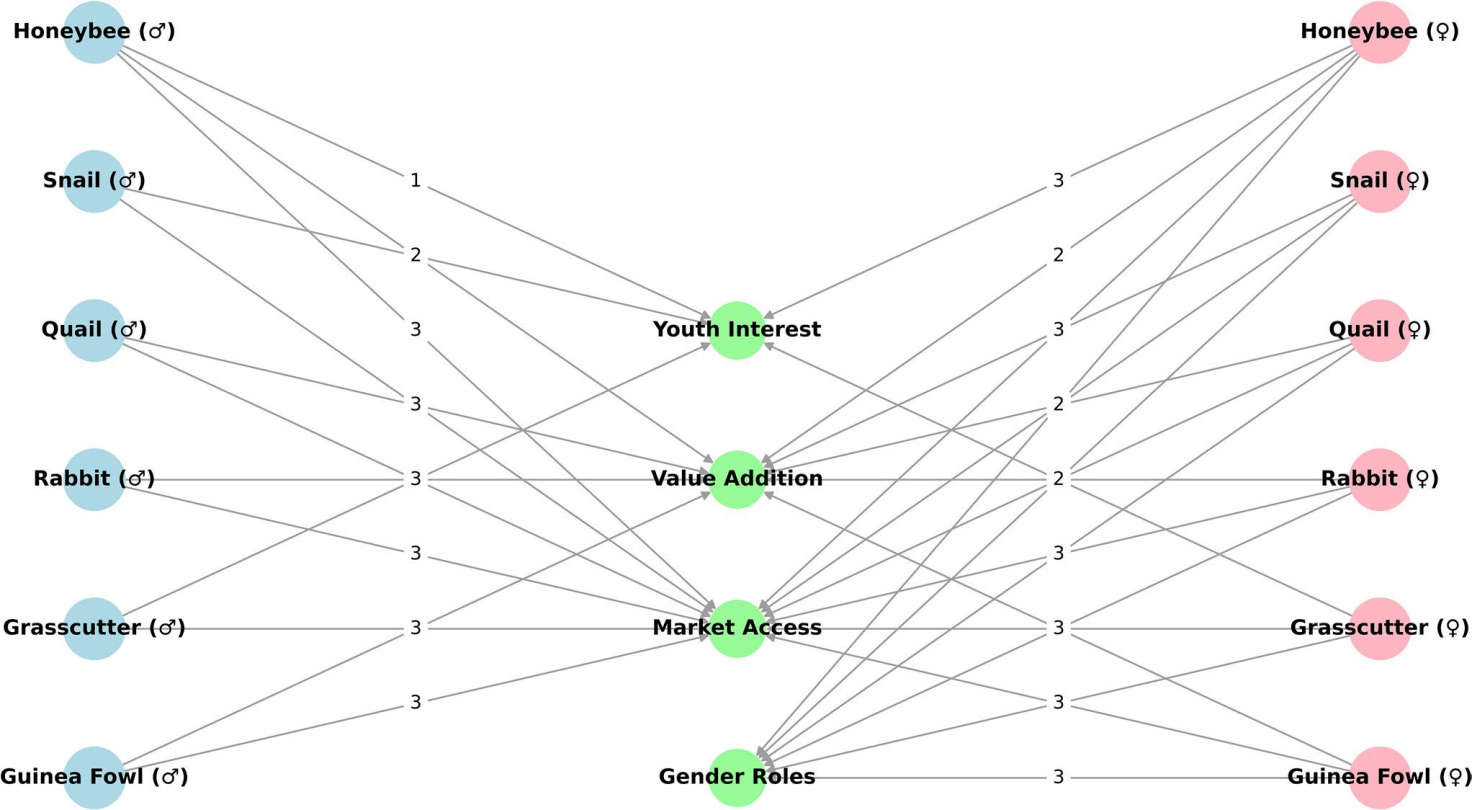
Gender-differentiated relationship map of microlivestock species and thematic priorities. This network diagram visualises the associations between six microlivestock species (disaggregated by gender) and four priority themes identified during FGDs. Male nodes (♂) appear in blue on the left, female nodes (♀) in pink on the right, and thematic priorities in green at the centre. Arrows represent directional linkages, with edge labels indicating ranked importance (3 = high priority, 2 = moderate, 1 = low)

Figure 2 illustrates the directional links between microlivestock species, FGD-identified priority themes, and gender groups. This structure highlights both shared and gender-specific priorities across value chains, offering insights for targeted intervention design.

### Stakeholder priorities and recommendations

Table 7 presents a synthesis of insights from the KIIs conducted, highlighting perceived constraints, emerging opportunities, and priority recommendations for value chain development. Irrespective of the value chain, poor access to finance and credit was a common constraint identified by all the key informants.

**Table 7:** Summary of insights from the KIIs

Species	Key Constraints	Opportunities	Recommendations
Rabbit	Limited access to high-quality breeding stock and markets, and regulatory hurdles for product certification	Strong consumer demand, no cultural taboos around rabbit meat	Conduct public awareness campaigns, strengthen regulatory frameworks, and promote rabbit meat in school feeding programmes
Grasscutter	Limited access to quality breeding stock and markets, poor veterinary support, inadequate feed supply	Youth interest, digital marketing and potential for value-added processing	Increase access to finance, capacity building (production and processing, e-commerce), promote gender-inclusiveness
Snail	Low productivity due to lack of quality breeding stock, lack of commercial feed	High demand and potential for export market	Provide training on production, Policy and regulatory framework for snail farming.
Quail	Low consumer demand due to limited awareness, absence of government policy support	Value addition and demand for high-value eggs and grilled meat	Nutrition messaging, Policy inclusion in livestock development programmes, and support for the formation of cooperatives
Guinea Fowl	Low literacy among farmers, poor hatchability of eggs and seasonality of production	Strong cultural significance and demand, potential for value-added processing	Tailored gender-inclusive training programs for production and marketing
Honeybee	Product adulteration, poor access to modern beekeeping tools, and a lack of product certification mechanisms	Export potential, primarily through product diversification	Introduce honey certification, increase advocacy for women’s involvement and encourage cooperatives

## Discussion

This study offers an exploratory and timely analysis of six underexplored microlivestock value chains (rabbits, grasscutters, snails, honeybees, guinea fowl, and quail) in Nigeria, providing a valuable addition to earlier country-level assessments (FMARD 2020; FRN 2024). Findings for all six species demonstrate that, although microlivestock are increasingly recognised for their socio-economic and nutritional benefits, their value chains remain fragmented, under-resourced, and gender-imbalanced, with women underrepresented in commercial-oriented activities in the value chains (Assan 2014a, b; Ayeni et al. 2023; Oguche et al. 2025). Our study shows that even in locations characterised by high microlivestock density, only a minority of respondents (10–33%) rely on these species as their primary livelihood source, highlighting their predominantly supplementary role. While such production may serve as a strategy for diversifying household income, part-time engagement is typically associated with small-scale productivity, which constrains investment incentives, limits enterprise growth, and reduces technical efficiency (Muzekenyi et al. 2021; Giger et al. 2022; Nano 2023).

Key production constraints, such as limited access to species-specific feed and veterinary services, align with the findings from previous reports that highlighted animal feed challenges and systemic gaps in animal health infrastructure (FRN 2024). Farmers reporting that they mostly treat their sick animals themselves, and the use of generic rather than species-specific inputs across the value chains, indicates a lack of tailored services for microlivestock production, processing and/or marketing. This can limit animal productivity and the performance of the value chains. Our findings reveal that microlivestock producers often rear multiple species. The livestock species most commonly reared alongside microlivestock were chickens and goats. This is consistent with the recent National Agricultural Sample Census (NASC), which identified these two species as the most common livestock among agricultural households in Nigeria (NBS 2024). The high proportion of guinea fowl farmers who are also cattle farmers suggests a link between this microlivestock value chain and pastoral and agro-pastoral systems. These patterns reflect farmers’ use of multiple production systems and the integration of microlivestock into traditional livestock systems.

Value addition emerged as a major theme in the study, highlighting its critical role in enhancing product development, supply, and market opportunities within specific animal value chains (Assan 2013). During the FGDs, value addition was prioritised by both male and female producers and is reflected in the gender-differentiated relationship map (Fig. 2). Despite its recognised importance, our findings show that value addition remains limited by infrastructure deficits and a lack of technical capacity and training among producers. These constraints not only hinder enterprise growth but also restrict the potential development of the value chains (Farmery et al. 2021). Strengthening value addition offers a strategic entry point for expanding and diversifying the value chain beyond the prevailing single-actor model, in which producers are burdened with multiple responsibilities (Trienekens 2011; Farmery et al. 2021). By promoting specialisation and strengthening the capacity of diverse actors, the value chain can become more efficient, inclusive, and resilient, creating opportunities for youth, women, and small-scale entrepreneurs (Alawode 2025).

As the study’s findings show, gender disparity exists across the entire microlivestock value chain, though it varies by species. Men were primarily involved in production-related decisions, while women were often relegated to support roles related to feeding, cleaning, artisanal processing, and small-scale retailing. This structural imbalance in roles and decision-making has been reported in other livestock systems worldwide, limiting women’s ability to benefit equally from value chain activities (FAO 2011; Kristjanson et al. 2014; FAO and AUC 2020; Elias et al. 2024). These findings support previous studies on the need for gender-sensitive and transformative strategies that extend beyond mere participation of women in agricultural value chains (Elias et al. 2024; Njiru et al. 2024). These strategies focus on addressing the underlying power dynamics influencing gender imbalances and gender-related productivity levels through targeted training, access to finance, and support for market integration (Elias et al. 2024; Njiru et al. 2024).

Another recurring theme in the study was youth interest, particularly among honeybee, snail, and grasscutter producers. Findings from both the FGDs and KIIs indicate that male groups prioritise youth involvement because of its relatively low entry barriers, quick production cycles, and expanding market opportunities accessible using digital tools. However, support structures for young people’s participation in the value chains remain inadequate. As evidenced in the study locations (Table 1), youths (15–29) and young adults (15–35 years) present potentially important demographics in the microlivestock value chains. They constitute about 27.1% and 32.5% of Nigeria’s population, respectively, and face unemployment rates of 42–53%, underscoring an urgent need for viable and sustainable livelihood pathways (NPC 2020; NESG 2021; FMBPN 2025; NiRA 2025). Nonetheless, analysis of respondent demographics reveals that the average age of all sampled microlivestock producers exceeds these national youth age brackets (FRN 2019; NBS 2020). This suggests a low level of engagement among youths and young people in these value chains. Microlivestock systems may present a strategic opportunity to empower this demographic, especially when combined with targeted investments in youth-led enterprise development, digital literacy, and inclusive innovation systems (Betcherman and Khan 2015; Nchanji et al. 2023).

Across the six value chains studied, market access was a primary concern highlighted by both male and female producers. A high percentage of farmers sampled in this study primarily engaged in direct sales and informal trade channels, signalling limited participation in formal markets. Also, levels of producer organisation and association membership were factors contributing to varied market access across the value chains. Low membership levels were observed among guinea fowl (10.0%), quail (17.0%), and snail (27.8%) farmers, which may limit bargaining power, influence exploitative pricing, weaken coordination, and restrict access to markets. Conversely, higher membership rates among rabbit (95.0%), honeybee (70.0%), and grasscutter (61.1%) producers indicate better networking and organisation, and potentially stronger collective capacity to access services and markets (Kalogiannidis et al. 2024). The impact of varying membership levels of producer associations on market access highlights the importance of strengthening cluster farming and formation of cooperatives, particularly for value chains where actors are poorly organised (Endalew et al. 2024; Khanal et al. 2024; Gidelew et al. 2025).

Finally, while policy frameworks such as NL-GAS (2025–2030) and NATIP (2022–2027) (FMARD 2022; Nkwocha 2025) list microlivestock among priority species, the sector currently lacks cohesive implementation strategies. Evidence from this study emphasises the need for species-specific guidelines, extension packages, financing tools and aggregation models to bridge the gap between micro, small and medium-scale enterprises and sustainable commercialisation.

In conclusion, this study was undertaken to address gaps in the research on microlivestock in Nigeria, wherein previous studies had focused on specific species and/or aspects rather than the value chain in its entirety. Our study aimed to provide a consolidated value chain analysis of all six nationally recognised microlivestock species in Nigeria. Using an exploratory mixed-methods design, the study combined quantitative surveys, gender-disaggregated focus group discussions, and key informant interviews across six purposively selected production hubs. The inclusion of guinea fowl and quail, alongside rabbits, grasscutters, snails, and honeybees, provides the first integrated overview of the subsector.

The findings indicate that microlivestock species represent viable entry points for inclusive livestock transformation in Nigeria, particularly for women and youth. Despite their acclaimed biological and economic advantages, the subsector faces persistent technical, institutional, and market barriers. Beyond Nigeria, our findings align with evidence from other sub-Saharan African countries, where microlivestock production is similarly characterised by small-scale enterprise, high levels of informality, limited access to specialised inputs and animal health services, and weak integration into formal markets. However, in more developed settings, particularly in Europe and North America, microlivestock production is embedded within institutional environments characterised by stronger regulatory oversight, food safety and quality assurance systems, environmental safeguards, and certification frameworks that support traceability and niche market development (FAO, 2020). Within this broader context, the Nigerian experience reflects not a lack of biological or economic potential, but gaps in service delivery, market organisation, and institutional support.

To address these issues, a coordinated, multi-stakeholder investment in infrastructure, knowledge systems, policy alignment, and value addition is necessary. This study recommends that the FMLD, in collaboration with its state-level counterparts, research institutions and private actors, prioritise the development of species-specific policies, certification systems, and extension protocols. Also, financial institutions such as the Bank of Agriculture and the Bank of Industry should design bespoke credit facilities for microlivestock enterprises, with fair access given to youths and women. This will encourage more women and young people to participate in the value chains. Furthermore, we propose development-oriented research to test scalable solutions for integrating microlivestock into Nigeria’s existing livestock policy framework.

### Limitations of the study

While the study provides new insights into Nigeria’s microlivestock value chains, it has certain limitations. The sample was small and purposively selected, limiting the statistical generalisability of the results. Therefore, the research findings should be interpreted as indicative rather than representative of the entire national population. Additionally, the study’s cross-sectional approach prevented the analysis of seasonal changes in productivity and income, which are particularly important for species such as snails and guinea fowl. Finally, the study was conducted in states with active producer associations, which might have excluded emerging and marginalised production areas. To improve representativeness, future research should explore longitudinal study designs and broader sampling methods.

## Supplementary Information

Below is the link to the electronic supplementary material.

Supplementary Material 1

Supplementary Material 2

## Data Availability

Requests for the study’s data may be directed to the corresponding author. The anonymised dataset will be made publicly available at the International Livestock Research Institute (ILRI) dataset portal (https://data.ilri.org/portal/) or other appropriate outlet.
