# Supplementary material for: An assessment of Nigeria’s microlivestock value chain: insights from six species
Source: Trop Anim Health Prod. 2026 Mar 14;58(3):174. doi: 10.1007/s11250-026-04960-8 (PMC12989003; doi:10.1007/s11250-026-04960-8)
Supplement: Supplementary file 1 — Supplementary Material 1 [file 11250_2026_4960_MOESM1_ESM.pdf]

An assessment of Nigeria's Microlivestock Value Chain: Insights from Six Species

Tropical Animal Health and Production

Dolapo Enahoro[1], Oladeji Bamidele[2,3*], Dare Akerele[4], Olusegun O. Ojebiyi[5], Adetunji O. Iyiola-Tunji[6], Wasiu A. Olaniyi[7], Joseph Karugia[3] and Isabelle Baltenweck[3]

[1]People, Policies and Institutions Program, International Livestock Research Institute, C/o IWMI-Ghana, Accra, Ghana.

[2]Department of Biological Sciences, Kings University, Odeomu, Osun State, Nigeria

[3]People, Policies and Institutions Program, International Livestock Research Institute, Nairobi, Kenya.

[4]Department of Agricultural Economics and Farm Management, Federal University of Agriculture, Abeokuta, Nigeria

[5]Department of Animal Nutrition and Biotechnology, Faculty of Agricultural Sciences, Ladoke Akintola University of Technology, Ogbomoso, Oyo State, Nigeria

[6]National Agricultural Extension and Research Liaison Services (NAERLS), Ahmadu Bello University, Zaria, Kaduna State, Nigeria

[7]Department of Animal Science, Adekunle Ajasin University, Akungba-Akoko, Ondo State, Nigeria.

*Corresponding author: bamideledeji@gmail.com.

Introduction

A structured, interviewer-administered questionnaire was developed to capture comparable, species-specific data across six microlivestock value chains (grasscutter, guinea fowl, honeybee, quail, rabbit, and snail). The instrument was organised into standardised modules to enable cross-species synthesis while retaining technical detail relevant to each enterprise.

The questionnaire covers five core sections: (A) socio-demographic characteristics and livelihood diversification, including co-ownership of other livestock; (B) input sourcing and service access (feeds, foundation/breeding stock, and animal health support); (C) enterprise production and husbandry parameters (housing/management system, reproduction and productivity indicators, stock size and structure, and performance metrics); (D) products, value chain roles, marketing channels, buyer characteristics, demand geography, and price or sales volumes; and (E) awareness of policy or institutional support relevant to the value chain. Most items are closed-ended (binary, categorical, and multiple-response), complemented by a limited number of numeric entries (e.g. stock size, age, prices, yields, production volumes). Species-specific wording was used where required (e.g. hive numbers and honey outputs for honeybee; clutch and hatchability measures for guinea fowl; kindling interval and pre-weaning mortality for rabbit), while preserving consistent constructs for comparative analysis.

The compiled questionnaire below presents the final instrument as implemented in the field.

1. GRASSCUTTER

Section A: Socio-demography

1. State:
2. Specific location (name of town and local government area) of your farm in the state:
3. Gender
    a) Female
    b) Male
4. Age ___________
5. Marital status
    a. Single
    b. Married
    c. Divorced
    d. Widow/Widower
6. Level of educational attainment
    a) No formal education
    b) Senior Secondary School Certificate/WASCE
    c) National Diploma
    d) First degree / HND
    e) Second degree
    f) PhD holder
7. Is grass cutter farming your sole occupation for income?
    a) Yes, that is all I do
    b) No, *I have other sources of income (e.g. crop farming, trade, civil servant)*
8. Which of these traditional livestock do you also keep? *(Multiple responses)*
    a. Cattle
    b. Sheep
    c. Goats
    d. Pigs
    e. Poultry
    f. None
9. What is the average total number of grasscutters you have on the farm________________,
    a. then follow up with the distribution: *Kid __________, Growers___________, Does________________, Male_____________________*---

10. Number of years of experience in grasscutter production
    a) < 1 years
    b) 1-2 years
    c) 3-5 years
    d) 5-10 years
    e) >10 years
11. Are you a member of a grasscutter farmers association/cooperative?
    a) Yes
    b) No

Section B: Input Supply to Grasscutter Value Chains

12. How do you make provision for feeds in your enterprise? *(Multiple responses)*
    a) I purchase commercial feeds
    b) I formulate my own feed
    c) Through Grasscutter farmers association/cooperative
    d) Through arrangement with other farmers
    e) I formulate feeds for my farm and other farmers
    f) Involved in wholesale distribution of commercial feeds
    g) I supplement with forages
13. How do you get grasscutter breeds/ stocks for your farm? *(Multiple responses)*
    a) Through commercial farmers

b) Recycling of stocks through fellow farmers
c) Through breeding stock suppliers
d) Through grasscutter farmers associations
e) Through breeding stock suppliers outside of Nigeria

14. How do you treat your animals for health issues? *(Multiple responses)*
    a) Veterinarian
    b) Para-vet/Community animal health workers
    c) Other farmers from the association/cooperative
    d) Self

Section C: Grasscutter Production Enterprise

15. Which of the following production systems does your enterprise best fit in? *(multiple responses apply)*
    a) Wooden cage system
    b) Metal cage system
    c) Floor or low fenced pen
    d) Concrete (two or three tiers) housing
    e) A combination of cage and floor system
16. What is the estimated kindling interval for grasscutter on your farm? [Kindling interval is the number of days between two successive deliveries or kindling by the doe]
    a) 5 - 6 months
    b) 7 - 8 months
    c) 9 – 10 months
    d) > 10 months
17. What is the mean litter size at birth (number of litters)
    a) < 3
    b) 4
    c) 5
    d) 6
    e) 7
    f) 8
    g) 9
    h) 10
18. What is the mean litter size at weaning?
    a) <3
    b) 4
    c) 5
    d) 6
    e) 7
    f) >8
19. What is the age of the litter at weaning?
    a) < 3 weeks
    b) 3 – 4 weeks
    c) 5 -6 weeks
    d) 7 - 8 weeks
20. What is the average **age** of stocks at market weight?
    a) 4-5 months
    b) 6-7 months
    c) 8-9 months
    d) 10 months
21. What is the average **weight** of stocks at market age?________________
22. Which type of breed do you farm? *(Multiple responses)*
    a) Normal grey-green
    b) Immaculate white
    c) Golden Brown

d) I do not know
e) Others (specify)

Section D: Products, by-products, markets

23. Which of the following businesses do you engage in? *(Multiple responses)*
    a. Sale of live grass cutters
    b. Processing and dressing of carcass into parts
    c. Processing and sale of breeding stock
    d. Sale of manure
    e. Sale of inputs (feed, cages)
24. To which of these groups do you sell your grasscutters (live/dressed)? *(Multiple responses)*
    a) middlemen
    b) Directly to consumers
    c) Restaurants, supermarkets
    d) Other farmers
25. Who (gender) do you sell most to?
    a) females
    b) males
    c) Equal
26. Which region of the country do you consider has the most demand (market) for grasscutter meat?
    a) North east
    b) North west
    c) North central
    d) South west
    e) South east
    f) South south
27. What is the market price of an adult (live) grasscutter (in Naria) _________
28. What is the profit made from the sale of an adult grasscutter (in Naira)_________

Section E: Policy

29. Are you aware of the federal government's policy on promoting grasscutter production?
    a) Yes
    b) No

2. GUINEA FOWL

Section A: Socio-demography

1. State:
2. Specific location (Name of town and local government area) of your farm in the state:
3. Gender
    c) Female
    d) Male
4. Age ___________
5. Marital status
    a. Single
    b. Married
    c. Divorced
    d. Widow/Widower
6. Level of educational attainment
    g) No formal education
    h) Senior Secondary School Certificate/WASCE
    i) National Diploma
    j) First degree / HND
    k) Second degree
    l) PhD holder
7. Do you raise guinea fowl as your sole occupation?
    c) Yes, that is all I do
    d) No, *I have other sources of income (e.g. crop farming, trade, civil servant)*
8. Which of these traditional livestock do you also keep? *(Multiple responses)*
    a. Cattle
    b. Sheep
    c. Goats
    d. Pigs
    e. Chickens
    f. None
9. What is your average flock size ________________. *Then follow up with the distribution*
    a. *Keets __________, Growers___________, cocks________________, hens___________*
10. How long have you been keeping guinea fowls?
    f) < 1 years
    g) 1-2 years
    h) 3-5 years
    i) 5-10 years
    j) >10 years
11. Are you a member of a group/association/cooperative on guinea fowl production?
    c) No
    d) Yes

Section B: Management System

12. Source of foundation stock (Tick as appropriate) a. Inherited [ ] b. Purchase from market [ ] c. Purchase from neighbour [ ] d. Borrowed [ ] e. Gift [ ] f. Others [ ]
13. Management practice (Tick as appropriate)
    a. Free range /extensive
    b. Semi-intensive
    c. Complete confinement (intensive)
14. Do you give supplementary feeds? Yes [ ]; No [ ].
15. Do you offer your birds drinking water? Yes [ ]; No [ ].
16. Who are the members of household responsible/in charge of feeding and offering water to the birds? (a) Father only (b) Mother only (c) children only (d) Father and children only (e) Mother and Children only (d) Others
17. Do you have access to veterinarians? Yes [ ]; No [ ].

18a. If yes, which category(ies) of veterinarians? *(Multiple responses)* (a) Government vet. [ ]
(b) Private vet. [ ] (c) Para-vet/community animal health worker
18b. If no, how do you treat your birds (a) Self medication [ ].(B) farmer group (c) both

Section C. Productivity

19. No of clutch of eggs laid in year…………………………………………………
20. No of eggs in a clutch……………………………………………………………
21. No of eggs that hatch in a clutch…………………………………………………….
22. No of keet mortality/year…………………………………………………………
23. No of growers mortality/year………………………………………………..
24. What season of the year do you record the highest mortality? (a) wet [ ] (b) Hot-dry [ ] . (c)
Harmattan [ ].

Section D: Products, by-products, markets

25. Which of the following businesses do you engage in? *(Multiple responses)*
    a. Sale of live birds
    b. Processing and dressing of carcass into parts
    c. Sale of eggs
    d. Sale of manure
    e. Sale of inputs (e.g. feed)
    f. None
26. To which of these groups do you sell your guinea fowl (live/dressed)? *(Multiple responses)*
    e) Middlemen/offtakers
    f) Directly to consumers
    g) Restaurants, supermarkets
27. Who (gender) do you sell most to?
    d) females
    e) males
    f) Equal
28. Which region of the country do you consider has the most demand (market) for guinea fowl?
    g) North east
    h) North west
    i) North central
    j) South west
    k) South east
    l) South south
29. What is the average price of a live guinea fowl (adult) (in Naria) _________
30. In the last 12 months, how many guinea fowls have you sold?_____________

3. HONEYBEE

Section A: Socio-demography

1. State:
2. Specific location (Name of town and local government area) of your bee farm in the state:
3. Gender
    e) Female
    f) Male

4. Age................................
5. Marital status
    a. Single
    b. Married
    c. Divorced
    d. Widow/Widower
6. Level of educational attainment
    m) No formal education
    n) Primary education
    o) Senior Secondary School Certificate/WASCE
    p) National Diploma
    q) First degree / HND
    r) Higher Degree
7. Is beekeeping your sole occupation for income?
    a.Yes, that is all I do
    b. No, *I have other sources of income (e.g. crop farming, trading, civil servant)*
8. Which of these traditional livestock do you also keep? *(Multiple responses where necessary)*
    a. Cattle
    b. Sheep
    c. Goats
    d. Pigs
    e. Chickens
    f. None
    g. Others (Specify)
9. How long have you been involved in beekeeping production?
    k) < 1 year
    l) 1-2 years
    m) 3-5 years
    n) 5-10 years
    o) >10 years
10. Are you a member of a beekeeping association/cooperative?
    b. Yes
    c. No

Section B: Production And Operations

11. What is your scale of production?
    a. Small scale
    b. Medium scale
    c. Large Scale
12. How many beehives do you manage?
    1. 1-10
    2. 11-50
    3. 51-100
    4. More than 100
13. How many liters of honey are harvested from each hive per season?
14. What is your role in Honey Value Chain (multiple responses apply)
    a. Beekeeper
    b. Input Supplier

c. Processor
d. Distributor
e. Retailer
f. Other (please specify)

15. What is your annual capacity for honey production?
    1. < 250 kg
    2. 250 - 500 kg
    3. 501 - 2,000 kg
    4. 2,001 - 5,000 kg
    5. More than 5,000 kg
16. What type(s) of honey do you produce (multiple responses apply):
    1. Wildflower
    2. Forest
    3. Citrus
    4. Savannah
    5. Other (please specify):
17. What is the duration (months) between the hive installation and colonization by honey bees?
18. What is the duration (months) between colonization and harvesting of honey?

Section C: Input Supply Sources

19. Where do you obtain your beekeeping inputs (e.g., beehives, protective gear, feeders)? (multiple responses apply)
    a. Local suppliers
    b. National suppliers
    c. International suppliers
    d. Self-produced
    e. Other beekeepers
    f. Cooperative/Association
20. What type of bee feed do you supply or use? (multiple responses apply)
    a. Sugar syrup
    b. Pollen substitutes
    c. Protein supplements
    d. Other (please specify)
21. Who are your main customers? (Select all that apply)
    a. Individual beekeepers
    b. Beekeeping cooperatives
    c. Honey processors
    d. Agricultural supply stores
    e. Other (please specify)

Section D: Markets

22. Which of the following products and by-products do you produce & sell? *(Multiple responses)*
    a. honey
    b. Bees wax
    c. Propolis
    d. Royal jelly
    e. Bees venom
23. To which of these groups do you sell your products/by-products? *(Multiple responses apply)*
    h) Middlemen
    i) Directly to consumers
    j) Restaurants, supermarkets
    k) Export
24. Who (gender) do you sell most to?
    g) females
    h) males
    i) Equal

25. Which region of the country do you consider has the most demand (market) for honeybee products?

m) North east
n) North west
o) North central
p) South west
q) South east
r) South south

26. What is the market price per litre of honey (in Naira) _________

27. *What other by-product(s) do you sell and its price? (optional)*

- *Product*_____________________*Price/unit*_____________________________
- *Product*_____________________*Price/unit*_____________________________
- *Product*_____________________*Price/unit*_____________________________

Section E: Policy

28. Are you aware of any government's policy on honeybee production?

c) Yes,
d) No

4. QUAIL

Section A: Socio-demography

1. State:
2. Specific location (Name of town and local government area) of your quail farm in the state:
3. Gender
    g) Female
    h) Male

4. Age................................
5. Marital status
    a. Single
    b. Married
    c. Divorced
    d. Widow/Widower
6. Level of educational attainment
    s) No formal education
    t) Primary education
    u) Senior Secondary School Certificate/WASCE
    v) National Diploma
    w) First degree / HND
    x) Higher Degree
7. Is quail farming your sole occupation for income?
    a.Yes, that is all I do
    b. No, *I have other sources of income (e.g. crop farming, trading, civil servant)*
8. Which of these traditional livestock do you also keep? *(Multiple responses where necessary)*
    a. Cattle
    b. Sheep
    c. Goats
    d. Pigs
    e. Chickens
    f. None
    g. Others (Specify)

9. How long have you been in quail production?
    p) < 1 year
    q) 1-2 years
    r) 3-5 years
    s) 5-10 years
    t) >10 years
11. Are you a member of a quail production association/cooperative?
    d. Yes
    e. No

Section B: Input Supply to Quail Value Chains

12. Are you aware of any special commercial feed for quail in your locality?
    a. Yes
    b. No
    If Yes, what is the brand of feed?…………………………………….
13. Regarding feeding and watering, which of these apply to your quail farm? *(Multiple responses)*
    h) I purchase commercial quail feeds
    i) I produce my own feed for my quail farm
    j) Through Quail Producers Association/Cooperative
    k) I use chicken feed.
    l) I produce commercial quail feeds for my farm and for other farmers
    m) I supplement with greens, grains, and insects

n) I provide automated watering systems
o) I use manual drinkers

14. How do you get quails to stock your farm? *(Multiple responses where necessary)*
    f) Through commercial commercial hatchery farms
    g) Through Research Institutes
    h) Recycling of stock through fellow farmers
    i) Through breeding stock suppliers
    j) Through quail breeders Associations
    k) Through breeding stock suppliers outside of Nigeria
15. How do you treat your quail for health issues? *(Multiple responses where necessary)*
    e) Veterinarian
    f) Para-vet/Community animal health workers
    g) Other farmers from the association/cooperative
    h) Self

Section C: Quail Production Enterprise

16. What is the total number of quails you have on the farm……………………………,
    Indicate flock size per category.
    Layer _________ *Growers* _____________*Chicks* ________________
    Males ___________________ *Parent stock* _________________
17. Which housing reflects your production system?
    f) Battery/Cage system (Metal)
    g) Wooden cage
    h) Deep litter/Floor system
    i) A combination battery/cage and floor system
18. What is the average total number of eggs produced per quail annually? _______________
19. How do you get your quail eggs incubated/hatched? *(multiple responses apply)*
    a. Commercial hatcheries
    b. Research institutes.
    c. Farmers' cooperative
    d. Brooding
    e. I do not hatch
20. What is the mean weight of your quails at hatching?________________
21. What is the age of the quails at point of lay (sexual maturity)?
    a. 4-5 weeks
    b. 6- 7 weeks
    c. 8-9 weeks
    d. 10-11 weeks
    e. >12 weeks
    f. 10 weeks and above
22. What is the total duration (months) for quail production after which the birds are culled due to low productivity? ___________________________
23. What is the percentage hen day egg production?
24. How long do you keep/rear your quails for meat production? (months)____________
25. What is the live weight of your quails at market age? ....
26. Which breed(s) of quail do you raise in your enterprise? *(multiple responses apply)*
    a. **Japanese/Pharaoh/Coturnix Quail**
    b. **Tuxedo/English white quail**
    c. **Texas A&M Quail**
    d. **Manchurian/Chinese Painted Quail**

Section D: Products, by-products, markets

28. Which of the following businesses do you engage in? *(Multiple responses)*
    f. Sale of live quails
    g. Dressing and Processing of carcass for meat
    h. Sale of eggs

i. Sale of quail manure
j. Sale of inputs (feed, drugs, brooded birds, breeding stock, etc.)

29. To which of these groups do you sell your quail products? *(Multiple responses apply)*
    l) Farmers
    m) Researchers/Project Students
    n) Middlemen
    o) Directly to consumers
    p) Restaurants, supermarkets
30. Who (gender) do you sell most to?
    j) females
    k) males
    l) Equal
31. Which region of the country do you consider has the most demand (market) for quails?
    s) North east
    t) North west
    u) North central
    v) South west
    w) South east
    x) South south
32. What is the market price per live adult quail (in Naira) _________
33. *What is the market price per egg or dozen eggs?* _____________________

Section E: Policy

29. Are you aware of any organization promoting/supporting quail production?
    e) Yes, If yes provide a name______________________
    f) No

5. RABBIT

Section A: Socio-demography

1. State:
2. Specific location (Name of town and local government area) of your rabbit farm in the state
3. Gender
   i) Female
   j) Male
4. Age ___________
5. Marital status
   a. Single
   b. Married
   c. Divorced
   d. Widow/Widower
6. Level of educational attainment
   y) No formal education
   z) Senior Secondary School Certificate/WASCE
   aa) National Diploma
   bb) First degree / HND
   cc) Second degree
   dd) PhD holder
7. Is rabbit farming your sole occupation for income?
   e) Yes, that is all I do
   f) No, *I have other sources of income (e.g. crop farming, trade, civil servant)*
8. Which of these traditional livestock do you also keep? *(Multiple responses)*
   a. Cattle
   b. Sheep
   c. Goats
   d. Pigs
   e. Poultry
   f. None
9. What is the average total number of rabbits you have on the farm________________,
   a. then follow up with the distribution: *Kit __________, Weaners___________, Does________________, Buck_____________________*---

10. Number of years of experience in the rabbit production enterprises
    u) < 1 years
    v) 1-2 years
    w) 3-5 years
    x) 5-10 years
    y) >10 years
11. Are you a member of a meat rabbit production association/cooperative?
    e) Yes
    f) No

Section B: Input Supply to Rabbit Value Chains

12. How do you make provision for feeds in your rabbit enterprise? *(Multiple responses)*
    p) I purchase commercial rabbit feeds
    q) I produce my own feed for my rabbit farm
    r) Through Rabbit Producers Association/Cooperative
    s) Through cooperative arrangement with rabbit farmers
    t) I produce commercial rabbit feeds for my farm and for other rabbit farmers
    u) Involved in wholesale distributor
    v) I supplement with forages
13. How do you get rabbit breeds/genetic stocks for your farm? *(Multiple responses)*
    l) Through commercial farmers
    m) Recycling of stocks through fellow farmers

n) Through breeding stock suppliers
o) Through Rabbit breeders Associations
p) Through breeding stock suppliers outside of Nigeria

14. How do you treat your rabbits for health issues? *(Multiple responses)*
    i) Veterinarian
    j) Para-vet/Community animal health workers
    k) Other farmers from the association/cooperative
    l) Self

Section C: Rabbit Production Enterprise

15. Which of the following production systems does your rabbit enterprise best fit in?
    j) Intensive cage rabbit system
    k) Floor system
    l) Pastured rabbit system
    m) A combination cage and floor system
16. What is the estimated kindling interval for rabbits on your farm? [Kindling interval is the number of days between two successive deliveries or kindling by the doe]
    e) < 50 days
    f) 66 days
    g) 73 days
    h) 80 days
    i) 87 days
    j) 94 days
17. What is the mean litter size at birth (kindling)
    i) 8 kits
    j) 7 kits
    k) 6 kits
    l) 5 kits
    m) 4 kits
18. What is the mean litter size at weaning?
    g) 7 weaners
    h) 6 weaners
    i) 5 weaners
    j) 4 weaners
    k) 3 weaners
19. What is the age of the litter at weaning?
    e) 28 days
    f) 35 days
    g) 42 days
    h) 49 days
    i) Varies
20. What is the trend for pre-weaning mortality i.e. what fraction of the kits in the litter die before weaning?
    a) 75%
    b) 50%
    c) 30%
    d) 25%
    e) 10%
21. What is the **age** of stocks at market weight.
    e) 2 months
    f) 2.5 months
    g) 3 months
    h) 3.5 months
    i) 4 months
22. What is the **weight** of stocks at market age?

a) 1.8 kg
b) 2.0 kg
c) 2.5 kg
d) 3.0 kg
e) >3.0 kg

23. Which of these breeds do you raise in your enterprise? *(Multiple responses)*
    f) Hyla
    g) Dutch
    h) Chinchilla
    i) New Zealand white
    j) Californian
    k) Flemish giant
    l) Harlequin
    m) Angora
    n) Checkered Giant
    o) Hyplus
    p) Hycole
    q) Crossbreed

Section D: Products, by-products, markets

24. Which of the following businesses do you engage in? *(Multiple responses)*
    a. Sale of live rabbit
    b. Processing and dressing of carcass into parts
    c. Processing and sale of skin
    d. Sale of rabbit manure
    e. Sale of inputs (feed, breeding stock, etc.)
25. To which of these groups do you sell your rabbit meat (live/dressed)? *(Multiple responses)*
    q) middlemen
    r) Directly to consumers
    s) Restaurants, supermarkets
26. Who (gender) do you sell most to?
    m) females
    n) males
    o) Equal
27. Which region of the country do you consider has the most demand (market) for rabbit meat?
    y) North east
    z) North west
    aa) North central
    bb) South west
    cc) South east
    dd) South south
28. What is the price per kg live weight of an adult rabbit (in Naria) _________
29. What is the profit per kg live weight of an adult rabbit (in Naira)_________

Section E: Policy

30. Are you aware of the federal government's policy on promoting rabbit production?
    g) Yes
    h) No

6. SNAIL

Section A: Socio-demography

1. State:
2. Specific location (Name of town and local government area) of your farm in the state:
3. Gender
   k) Female
   l) Male
4. Age ___________
5. Marital status
   a. Single
   b. Married
   c. Divorced
   d. Widow/Widower
6. Level of educational attainment
   ee) No formal education
   ff) Senior Secondary School Certificate/WASCE
   gg) National Diploma
   hh) First degree / HND
   ii) Second degree
   jj) PhD holder
7. Is snail farming your sole occupation for income?
   g) Yes, that is all I do
   h) No, *I have other sources of income (e.g. crop farming, trade, civil servant)*
8. Which of these traditional livestock do you also keep? *(Multiple responses)*
   a. Cattle
   b. Sheep
   c. Goats
   d. Pigs
   e. Poultry
   f. None
9. What is the scale of production of your snail farm?
   a. Small scale
   b. Medium scale
   c. Large scale

10. What is the total number of snails (stock size) you have on the farm:
   a. <200, 201-400, 401-400, 601-800, 801 – 1000, >1000
   b. If possible, then follow up with the distribution: *hatchling/baby snail __________, Growers___________, breeder/matured snails________________,*

11. Number of years of experience in the snail production enterprises
   z) < 1 years
   aa) 1-2 years
   bb) 3-5 years
   cc) 5-10 years
   dd) >10 years
12. Are you a member of a snail farmers association/cooperative?
   g) Yes
   h) No

Section B: Input Supply to Snail Value Chains

13. How do you make provision for feeds in your snail enterprise? *(Multiple responses)*
   w) I purchase commercial feed
   x) I produce my own compounded feed
   y) I feed them with household/kitchen waste
   z) I use vegetables and fruits
   aa) I supplement with calcium

14. How do you source for foundation stock? *(Multiple responses)*
    q) Purchase at local market
    r) Hunting in the wild
    s) Gift
    t) Through snail farmers associations
15. How do you treat your snails for health issues? *(Multiple responses)*
    m) Veterinarian
    n) Para-vet/Community animal health workers
    o) Other farmers from the association/cooperative
    p) Self
    q) I do not treat

Section C: Snail Production Enterprise

16. Which of the following housing system(s) do you use? *(Multiple responses)*
    n) Baskets
    o) Drums
    p) Used tyres
    q) Constructed pens
17. What is the estimated no of clutch per year for a snail? (how many times can it lay eggs in a year)
    k) 1 time
    l) 2 times
    m) 3 times
    n) 4 times
    o) 5 times
    p) >5 times
18. What is the mean size of a clutch of eggs laid per snail?
    n) <10
    o) <11- 20
    p) 21 - 30
    q) 31- 40
    r) 41-50
19. At what age (month) do the hatchlings become growers?__________________
20. At what age (months) do the growers become mature (adult) for sale?___________________
21. Which of these species do you keep? *(Multiple responses)*
    r) Archachatina marginata
    s) Achatina achatina
    t) Achatina fulica
    u) Limicolaria sp
    v) I don't know

Section D: Products, by-products, markets

22. Which of the following businesses do you engage in? *(Multiple responses)*
    a. Sale of live snails
    b. Processing and dressing of snails
    c. Sale of the shells
    d. Sale of inputs (feed, breeding stock, etc.)
23. To which of these groups do you sell your snails (live/dressed)? *(Multiple responses)*
    t) middlemen
    u) Directly to consumers
    v) Restaurants, supermarkets
    w) Exporters
24. Who (gender) do you sell most to?
    p) females
    q) males

r) Equal

25. Which region of the country do you consider has the most demand (market) for snails?
    ee) North east
    ff) North west
    gg) North central
    hh) South west
    ii) South east
    jj) South south
26. What is the average selling price of an adult snail (in Naria) _________
27. What is the estimated profit made from the sale of an adult snail (in Naira)_________

Section E: Policy

28. Are you aware of the federal government's policy on promoting snail production?
    i) Yes
    j) No
