# Supplementary material for: An assessment of Nigeria’s microlivestock value chain: insights from six species
Source: Trop Anim Health Prod. 2026 Mar 14;58(3):174. doi: 10.1007/s11250-026-04960-8 (PMC12989003; doi:10.1007/s11250-026-04960-8)
Supplement: Supplementary file 2 — Supplementary Material 2 [file 11250_2026_4960_MOESM2_ESM.pdf]

An assessment of Nigeria's Microlivestock Value Chain: Insights from Six Species

Tropical Animal Health and Production

Dolapo Enahoro[1], Oladeji Bamidele[2,3*], Dare Akerele[4], Olusegun O. Ojebiyi[5], Adetunji O. Iyiola-Tunji[6], Wasiu A. Olaniyi[7], Joseph Karugia[3] and Isabelle Baltenweck[3]

[1]People, Policies and Institutions Program, International Livestock Research Institute, C/o IWMI-Ghana, Accra, Ghana.

[2]Department of Biological Sciences, Kings University, Odeomu, Osun State, Nigeria

[3]People, Policies and Institutions Program, International Livestock Research Institute, Nairobi, Kenya.

[4]Department of Agricultural Economics and Farm Management, Federal University of Agriculture, Abeokuta, Nigeria

[5]Department of Animal Nutrition and Biotechnology, Faculty of Agricultural Sciences, Ladoke Akintola University of Technology, Ogbomoso, Oyo State, Nigeria

[6]National Agricultural Extension and Research Liaison Services (NAERLS), Ahmadu Bello University, Zaria, Kaduna State, Nigeria

[7]Department of Animal Science, Adekunle Ajasin University, Akungba-Akoko, Ondo State, Nigeria.

*Corresponding author: bamideledeji@gmail.com.

Introduction

The qualitative data collection instruments presented in this appendix were developed to support an exploratory, mixed-methods assessment of microlivestock value chains in Nigeria. The Focus Group Discussion (FGD) guide was designed to elicit collective perspectives from producers on production practices, constraints, opportunities, gender dynamics, market participation, and capacity-building needs across microlivestock enterprises. During data collection, the species under discussion was selected as appropriate for each study location, including grasscutter, guinea fowl, honeybee, quail, rabbit, and snail. A common question structure was retained across species to enable cross-value-chain comparison while allowing for species-specific contextualisation.

FGDs were conducted separately for men and women to capture gender-differentiated experiences, priorities, and constraints along the value chain. The guide combines open-ended discussion with participatory ranking exercises to identify priority challenges, opportunities, and strategies, as well as areas of consensus and divergence among participants.

The Key Informant Interview (KII) guide was designed to complement the FGDs by capturing expert and institutional perspectives on the structure, performance, constraints, opportunities, and policy and regulatory environment of the selected microlivestock value chains. KIIs targeted representatives of producer organisations, extension and research institutions, and other stakeholders with in-depth sector

knowledge. Together, the FGD and KII guides provided triangulated qualitative evidence to contextualise survey findings and inform species-specific and cross-cutting recommendations. The two guides are presented below:

I. Focus Group Discussion (FGD) Guide

Part A: Production, Feed, and Animal Health

A1. Value Chain Mapping

1. What activities and actors are involved in the (select as appropriate: grasscutter/guinea fowl/ honeybee/quail/rabbit/snail) value chain in your community?
2. Have all relevant actors been captured (e.g. producers, input suppliers, traders, processors, middlemen/women)?
   Participants list actors and describe their roles.

A2. Production Challenges

3. What are the main challenges you face in select as appropriate: grasscutter/guinea fowl/honeybee/quail/rabbit/snail) production?
   Rank these challenges in order of importance based on their effect on business performance. Why were these challenges ranked in this order?

A3. Opportunities for Improvement

4. What opportunities exist to improve (select as appropriate: grasscutter/guinea fowl/ honeybee/quail/rabbit/snail) production and business performance?
   Rank these opportunities based on their ease of implementation. Provide reasons for the ranking.

A4. Solutions and Strategies

5. What strategies can be used to address the top three challenges identified?
6. Who are the key actors required for each strategy to succeed (e.g. farmers, government, extension agents, private sector)?
   Rank the importance of these actors for each strategy.

Part B: Gender and Market Access

B1. Participation of Women and Youth

7. How do women and youth currently participate in (select as appropriate: grasscutter/guinea fowl/ honeybee/quail/rabbit/snail) production, processing, and marketing?
8. In which activities are they underrepresented, and why?

B2. Market Access and Value Addition

9. What specific barriers do (select as appropriate: grasscutter/guinea fowl/ honeybee/quail/rabbit/snail) producers face in selling and marketing their products?
   Consider economic, cultural, gender-related, and institutional barriers. Rank these barriers according to how severely they limit market access.

10. How should government or other agencies address each barrier, and why?

Part C: Economic and Social Impact

Livelihood Contributions

11. Compared with traditional livestock (e.g. cattle, sheep, chickens), what does (select as appropriate: grasscutter/guinea fowl/ honeybee/quail/rabbit/snail) production contribute to household livelihoods?
    Rank these contributions in order of importance.
12. Share specific experiences illustrating how (select as appropriate: grasscutter/guinea fowl/ honeybee/quail/rabbit/snail) production has affected household income or wellbeing.

Part D: Public Engagement and Capacity Building

D1. ICT and Social Media Use

13. Do farmers in this group use ICT tools or social media for their (select as appropriate: grasscutter/guinea fowl/honeybee/quail/rabbit/snail) business?
14. Does this reflect the wider community experience?
15. What ICT tools or platforms are used, and for what purposes?
    Rank these tools based on their importance for business expansion.
16. What challenges limit the use of ICT and social media in (select as appropriate: grasscutter/guinea fowl/ honeybee/quail/rabbit/snail) businesses?
    Rank these challenges based on severity and explain the reasons.

D2. Capacity Building Needs

17. What types of training are most needed by (select as appropriate: grasscutter/guinea fowl/ honeybee/quail/rabbit/snail) producers?
    Rank training needs based on importance for improving business performance.
    Explain the reasons for the ranking.

II. Key Informant Interview (KII) Guide

1. How would you describe the current state of (select as appropriate: grasscutter/guinea fowl/ honeybee/quail/rabbit/snail) production in Nigeria?
2. Who participates more in the (select as appropriate: grasscutter/guinea fowl/ honeybee/quail/ rabbit/snail) value chain (men or women), and in which activities?
   What factors explain this pattern?
3. What changes are required to promote greater participation of women and youth?
4. What are the major challenges facing (select as appropriate: grasscutter/guinea fowl/ honeybee/quail/rabbit/snail) farmers with respect to:
   - Production
   - Value addition
   - Market access

  - Finance
  - Product demand

  Are these challenges experienced differently by men and women? If yes, how?

5. What existing or emerging opportunities exist in (select as appropriate: grasscutter/guinea fowl/honeybee/quail/rabbit/snail) production, processing, and marketing?
   - Consider innovation, equipment, and technology.

     What is needed to enable men, women, and youth to benefit from these opportunities?
6. How do farmers currently finance the start-up or expansion of (select as appropriate: grasscutter/guinea fowl/honeybee/quail/rabbit/snail) enterprises?
   What alternative financing mechanisms could be explored?
7. What policies, regulations, or institutional frameworks affect the (select as appropriate: grasscutter/guinea fowl/honeybee/quail/rabbit/snail) value chain?
   Are there cultural norms or practices that also influence participation?
8. Can you describe any successful or ongoing initiatives supporting (select as appropriate: grasscutter/guinea fowl/honeybee/quail/rabbit/snail) value chain development? Who implemented them, and what lessons can be drawn?
